# Practical Notes on Diagnosis and Treatment

**Published:** 1910-05-28

**Authors:** 


### Practical Notes on Diagnosis and Treatment.

**Otitis Media.**

Both quinine and salicylates should be avoided in treating patients affected with acute disease of the middle ear, as permanent impairment of hearing may be caused.—*Dr. Brand.*

**Infantile Œdema.**

The common cause of œdema of the feet and legs in infancy is the malnutrition resulting from chronic wasting disorders, such as tuberculosis, syphilis, prolonged alimentary affections, and any cause of marasmus. Sometimes it is due to toxæmia or to renal mischief.

**Dry Labour.**

When the amniotic fluid has escaped prematurely, and the os uteri remains contracted, or where there is hyperplasia of the cervix with rigidity, a solution of cocaine, 10 grains to the ounce, applied to the os and cervix, acts very efficiently in relaxing the spasm; while some anæsthetic effect will also be produced upon the mucous membrane of the vagina, minimising the discomfort of the passage of the head.

**Foreign Body in the Ear.**

An excellent method of removing foreign bodies from the ear or nose is that of suction. A piece of rubber tubing is attached firmly to the nozzle of a brass syringe. This is inserted into the meatus and brought in contact with the foreign body; by withdrawing the piston a vacuum is created and the body will be drawn into or against the free end of the tubing, and so may be got out. It is advantageous to moisten the end of the tubing with glycerine.—*Dr. C. A. Sturrock.*

**Dentition and the Ear.**

As a considerable part of the blood supply of the membrana tympani is derived from a branch of the internal carotid, and as the nervi vasorum of the carotid plexus and the inferior dental nerve both communicate with the otic ganglion, there is thus a connection between the teeth and the blood supply of the tympanic membrane. An erupting or a carious tooth may therefore be associated with hyperæmia of the drumhead. In any case of illness during dentition one should examine the ears as a matter of routine.—*Dr. A. T. Brand.*

**Restoration of Nerve Function.**

It is of the greatest importance to bring into play every means of favouring incoming and outgoing stimuli; to this end, the patient must be carefully instructed to assist the operator, making passive movements, by concentrating his attention on attempting to perform the same movements. Foot drop must be avoided by taking off the pressure and weight of the bed-clothes. The placing of the feet against a board while lying in bed, and the daily placing of the feet on the ground, in the effort to feel the weight of the body supported by the limbs, all tend to open up the old paths, or to restore function by new paths.—*Dr. F. W. Mott.*

**Ethmoid Suppuration.**

In dealing with obstinate cases of supra-orbital neuralgia the possibility of suppuration in the ethmoid cells should always be kept in mind.

**Earache in Children.**

The tympanic membrane should always be examined in all cases of indefinite pain in the head in young children, who are often quite unable to localise the pain of earache.

**Enteric Fever.**

Except in children, one should rarely, if ever, diagnose typhoid fever without seeing the spots. These are to be found in over 90 per cent. of adult cases.—*Dr. J. Abercrombie.*

**Pityriasis Rosea.**

This eruption is rapidly amenable to treatment. The patient should be soaked daily for half an hour in a bath of weak permanganate, after which salicylic vaseline (5 per cent.) is freely applied to the skin.—*Dr. A. Jamieson.*

**Diabetes Insipidus.**

The hygienic treatment of this condition involves attention to the state of the skin, warm clothing, and avoidance of chill. The diet should be as far as possible free from salt and poor in nitrogenous constituents. Treatment by drugs is unsatisfactory; ergot, opium, valerian, and gallic acid have been tried. No restriction should be placed on the amount of fluid taken.—*Dr. R. Hutchinson.*

**Pulmonary Tuberculosis.**

The differences in clinical course are so remarkable that he who can discriminate wisely between the different types, prognostically and therapeutically, has amply established his claim as a physician. Too much stress may be laid upon the bacteriological report and too scanty a consideration given to the clinical aspect. The prognosis remains correspondingly vague, and the therapeutic programme is often similarly limited and barren.—*Dr. R. W. Philip.*

**Diagnosis of Coma.**

It is of primary importance in cases of coma to ascertain if the patient can be roused, and the most effective stimulus for this purpose is firm and deep pressure on the supra-orbital nerves, by getting the thumb-nail into the supra-orbital notch. If no effect is produced by this method, you may take it for granted that the case is more serious than alcoholic coma alone.—*Mr. Chas. Gibbs.*

**Palpation in the Warm Bath.**

After immersion of about 15 minutes a great relaxation of the abdominal muscles occurs, and the result of palpating the patient in a hot bath is almost as satisfactory as under an anæsthetic. It has proved useful in the diagnosis of obscure pelvic or abdominal conditions, and has the advantage of co-operation on the part of the patient.—*Dr. J. R. Keith.*

